# Oculocardiac Reflex During Peribulbar Block: A Case Emphasizing the Role of Monitoring in Early Recognition

**DOI:** 10.7759/cureus.106903

**Published:** 2026-04-12

**Authors:** Leena Sekar, Sri Hari Vignesh R

**Affiliations:** 1 Department of Ophthalmology, Sri Lakshmi Narayana Institute of Medical Sciences, Puducherry, IND; 2 Department of Anesthesiology, Palakkad Institute of Medical Sciences, Palakkad, IND

**Keywords:** bradycardia, cataract surgery, oculocardiac reflex, peribulbar block, regional anesthesia

## Abstract

The oculocardiac reflex is a well-recognized autonomic phenomenon characterized by bradycardia in response to stimulation of ocular structures. It is most frequently encountered during strabismus surgery but may also occur during regional anesthesia techniques such as peribulbar block. We describe the case of a 64-year-old woman with no known comorbidities who developed sudden bradycardia during administration of a peribulbar block for cataract surgery. Following injection of 3 mL of 2% lignocaine with adrenaline (1:200,000) combined with hyaluronidase, a rapid decrease in heart rate (HR) was observed. The injection was immediately discontinued, and the patient was closely monitored. Although atropine was kept ready, it was not administered, as the HR returned spontaneously to baseline within 30 seconds. After confirming hemodynamic stability and adequacy of the block, surgery was completed uneventfully. This case highlights the importance of continuous monitoring and preparedness during a peribulbar block, even in routine clinical settings.

## Introduction

The oculocardiac reflex is a trigeminal-vagal reflex that manifests as a decrease in heart rate (HR) greater than 20% of baseline following stimulation of ocular or periocular structures. It is most commonly described during strabismus surgery, where traction on extraocular muscles serves as a well-established trigger [1]. Beyond surgical manipulation, the reflex may also be elicited during regional anesthesia, including peribulbar blocks, as well as by globe pressure or orbital instrumentation, with a prevalence of 14%-90% in strabismus surgery [2,3]. Although less frequently reported in these contexts, such occurrences remain clinically important due to the potential for abrupt cardiovascular changes. The peribulbar block is widely used in cataract surgery due to its effectiveness and favorable safety profile. Contemporary trends in ophthalmic anesthesia emphasize safer and more effective regional techniques, with peribulbar block commonly used for cataract procedures, and ongoing advances further improving its reliability and patient comfort [4]. Typically, 3-5 mL of local anesthetic is injected into the inferior peribulbar space at a standard anatomical location. However, despite its routine nature, reflex-mediated events can still occur. The reflex is mediated via the ophthalmic division of the trigeminal nerve as the afferent limb and the vagus nerve as the efferent limb, resulting in increased parasympathetic output and bradycardia. In many clinical settings, peribulbar blocks are performed in preoperative areas where monitoring practices may vary. This case highlights the occurrence of a significant oculocardiac reflex during routine peribulbar block in a stable patient despite standard technique, underscoring the need for vigilance even during seemingly low-risk ophthalmic regional anesthesia.

## Case presentation

**Figure 1:**
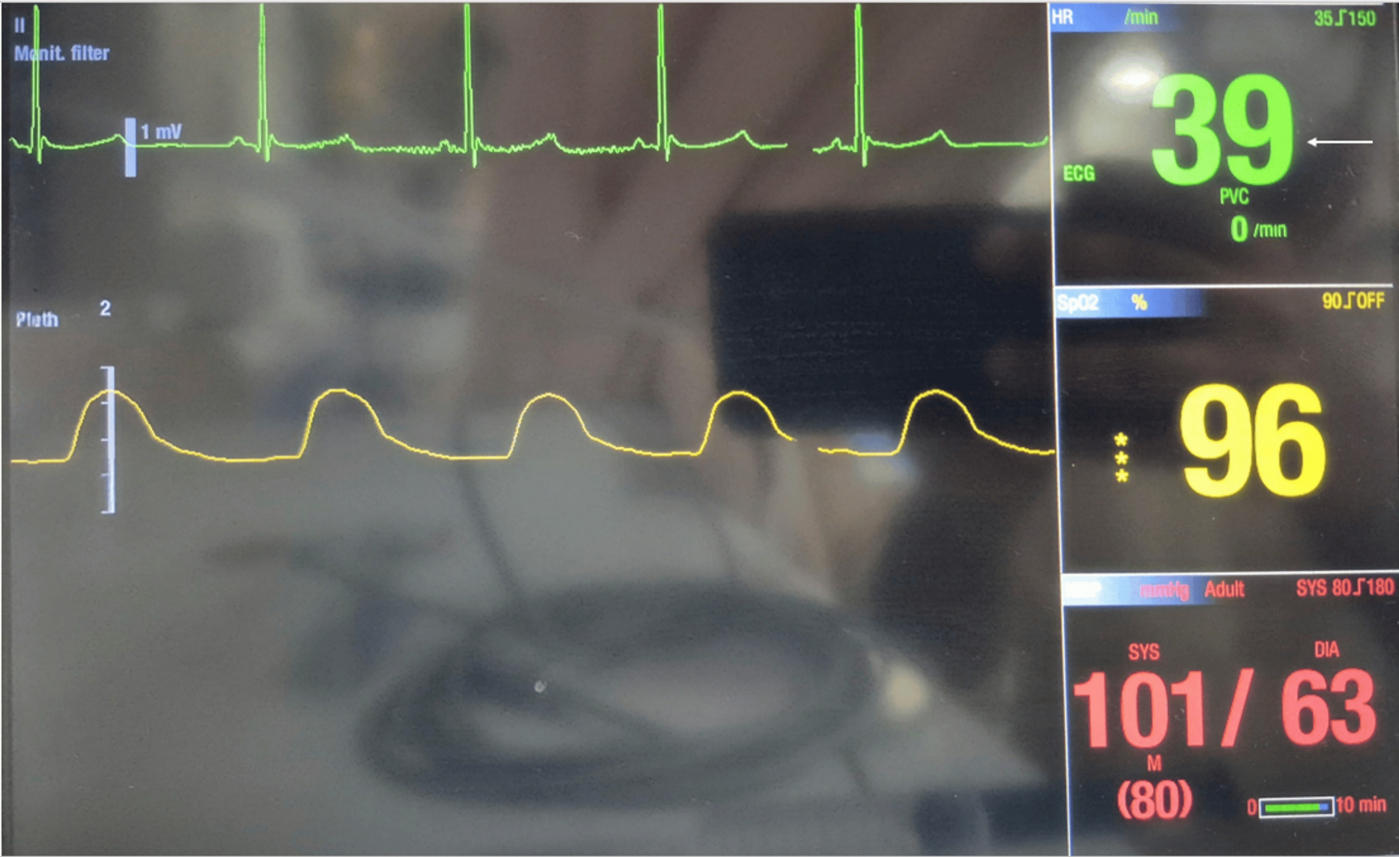
Monitor showing sudden-onset bradycardia approximately 10 seconds after the initiation of peribulbar injection, with heart rate decreasing from baseline 66 bpm to 39 bpm (arrow)

A 64-year-old woman with no known comorbidities was scheduled for elective cataract surgery. Preoperative evaluation was within normal limits. No sedative premedication was administered, and the patient remained calm and cooperative prior to block administration. An intravenous line was secured as part of standard preparation for all cataract procedures. A peribulbar block was administered under aseptic precautions in the preoperative area, with the patient in the sitting position. Monitoring included non-invasive blood pressure (NIBP), HR, and pulse oximetry (SpO₂). Baseline vital parameters were HR 66 bpm, BP 101/63 mmHg, and oxygen saturation 98% on room air. A total of 3 mL of 2% lignocaine with adrenaline (1:200,000) combined with hyaluronidase (50 IU/mL) was injected using a 24-G, 1-inch needle at the junction of the medial two-thirds and lateral one-third of the inferior peribulbar space at a depth of 1.5-2 cm. This volume is within the routinely used range of 3-5 mL. During injection, a sudden decrease in HR was observed, falling from 66 bpm to 39 bpm (Figure 1).

**Figure 2:**
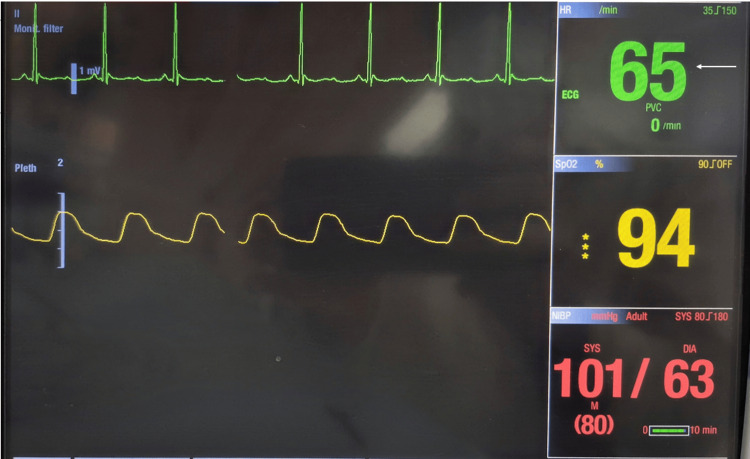
Monitor showing return of heart rate to baseline of 65 beats per minute (arrow) in approximately 30 seconds following immediate cessation of injection, indicating spontaneous resolution of the oculocardiac reflex

The patient remained conscious but exhibited signs consistent with a vagal response. The injection was immediately stopped. Atropine had been preloaded and kept ready as part of routine emergency preparedness; however, it was not administered, as the HR began to recover spontaneously. Within approximately 30 seconds, the HR returned to baseline levels (Figure 2).

Vital parameters, including NIBP, HR, and SpO₂, remained stable thereafter. The adequacy of the block was assessed and confirmed by the absence of extraocular movements, adequate globe akinesia, eyelid relaxation, and loss of corneal sensation. Given stable hemodynamics and satisfactory block, surgery was proceeded with. Cataract extraction was completed uneventfully, with no recurrence of bradycardia during the intraoperative or immediate postoperative period.

## Discussion

The oculocardiac reflex is mediated through a well-defined neural pathway involving both trigeminal and vagal components. The afferent limb arises from the ophthalmic division of the trigeminal nerve, transmitting sensory input from ocular structures to the brainstem. These signals are then relayed through interneuronal connections to the vagal motor nucleus, forming the efferent pathway. Activation of the vagus nerve leads to increased parasympathetic tone, resulting in bradycardia at the level of the sinoatrial node [2,3]. Although the reflex is most frequently associated with strabismus surgery, its occurrence during peribulbar block is less commonly emphasized in routine practice [5]. The clinical expression of this reflex can vary considerably. While mild bradycardia is most common, more pronounced responses, including arrhythmias or even transient cardiac arrest, have been reported depending on the intensity of the stimulus and patient-specific factors [6]. Standard monitoring during ophthalmic regional anesthesia should include continuous HR, pulse oximetry, and intermittent NIBP measurement to facilitate early recognition of reflex-mediated hemodynamic events. In the context of regional anesthesia, potential triggers include mechanical stimulation within the orbit, increased intraorbital pressure during injection, or needle-related factors. Even when standard techniques and drug volumes are used, as in this case, the reflex may still be elicited. Other potential causes of intra-procedural bradycardia, including vasovagal response, intravascular injection, and local anesthetic systemic toxicity, were considered less likely because of the immediate association with orbital stimulation, absence of neurological manifestations, and rapid spontaneous recovery after stopping the injection.

The present episode represented a marked oculocardiac reflex response, with an approximately 41% reduction in HR from baseline, despite spontaneous resolution following cessation of the triggering stimulus. An important aspect of this case was the prompt recognition of the event and immediate cessation of the triggering stimulus. The HR returned to baseline within a short duration without the need for pharmacological intervention. Although atropine was readily available, it was not required, reflecting an appropriate and measured clinical response. Pharmacologic intervention was withheld because the reflex resolved promptly after removal of the triggering stimulus, consistent with standard management principles that prioritize cessation of stimulation before administration of anticholinergic therapy. Following stabilization, reassessment confirmed adequate akinesia, absence of extraocular movements, eyelid relaxation, and effective sensory blockade. With stable vital parameters, including HR, BP, and oxygen saturation, proceeding with surgery was considered safe. This case also highlights a broader practical consideration. Peribulbar blocks are often performed in environments where monitoring may not be uniformly applied. The ability to detect and respond to such reflex events depends heavily on the availability of basic monitoring. Early recognition allows simple measures, such as stopping the stimulus, to be effective in preventing progression to more serious complications. Preventive strategies described in the literature include gentle injection technique, avoidance of excessive orbital pressure, slow administration of anesthetic solution, and readiness with anticholinergic agents in high-risk patients [5,6]

Overall, this case reinforces three important points.

First, the oculocardiac reflex may be triggered not only during surgical manipulation but also during the administration of a peribulbar block.

Second, continuous monitoring during block administration is essential, as early detection of bradycardia depends on real-time observation of vital parameters.

Third, the presence of an anesthetist or adequately trained personnel, even in procedures performed under local anesthesia, plays a crucial role in ensuring patient safety and timely intervention.

## Conclusions

Oculocardiac reflex can occur during peribulbar block even when standard techniques are used and in patients without comorbidities. Although this represents a single-case observation, it reinforces that routine monitoring, early recognition, and preparedness are essential for safe management. Careful reassessment following recovery allows safe continuation of surgery.
